# Wisdom and wellbeing in polish older adults: the mediating role of forgiveness

**DOI:** 10.3389/fpsyg.2023.1163113

**Published:** 2023-09-14

**Authors:** Paweł Brudek, Stanisława Steuden, Kinga Kaleta

**Affiliations:** ^1^Department of Psychology, John Paul II Catholic University of Lublin, Lublin, Poland; ^2^Department of Psychology, University of Economics and Human Sciences, Warsaw, Poland; ^3^Department of Psychology, Jan Kochanowski University of Kielce, Kielce, Poland

**Keywords:** wisdom, forgiveness, wellbeing, older adult, positive aging

## Abstract

**Objectives:**

Possible correlations between wisdom and wellbeing among older people have been extensively debated in psychology. At the same time, researchers emphasize that the effect of older adults' wisdom on their wellbeing depends on numerous mediators. A review of the literature suggests that forgiveness might be one such variable. Thus, this study aimed to investigate whether forgiveness mediated the relationship between wisdom and wellbeing in late adulthood.

**Method:**

In total, 481 participants aged from 60 to 92 years (*M* = 68.84; *SD* = 6.31) were involved in the study. All older people participating in the study lived independently in their households. Four psychological instruments were used: (1) the Self-Assessed Wisdom Scale; (2) the Heartland Forgiveness Scale; (3) the Psychological Wellbeing Scale; and (4) the Satisfaction with Life Scale. Therefore, the measurement of the analyzed variables was self-reported.

**Results:**

The results showed that forgiveness mediated the relationships between wisdom and wellbeing in the older population. The indirect effects demonstrated that forgiveness mediated the relationships between wisdom and wellbeing. Wisdom related to higher forgiveness (β = 0.21; *p* < 0.01), which, in turn, was related to a higher level of psychological (β = 0.48; *p* < 0.01) and subjective (β = 0.36; *p* < 0.01) wellbeing.

**Conclusion:**

These findings suggest that forgiveness is an important element of wisdom and wellbeing. The greater the wisdom the participants showed, the stronger the tendency to forgive and the better wellbeing they reported.

## Introduction

Wellbeing (Allen, 2008; Steptoe et al., 2015; Krok et al., 2021) is considered to be a central outcome of aging well (Sancho et al., 2020; Ren et al., 2021; Pocock et al., 2023). It may be conceptualized in the hedonic tradition as subjective wellbeing (SWB) or viewed from the eudaimonic perspective as psychological wellbeing (PWB) (Ryan and Deci, 2001). These two approaches capture different perspectives of a person's life. Subjective wellbeing is defined as maximizing pleasure and avoiding pain and embracing the evaluation of life as a whole, life satisfaction, happiness, positive affect, and the absence of negative mood (Diener et al., 2018). Psychological wellbeing, in turn, is understood as self-realization and being a fully functioning person. Psychological wellbeing is associated with self-acceptance, positive relations with others, a sense of autonomy, environmental mastery, purpose in life, and an orientation toward continued personal growth (Ryff, 1989). Previous research has shown that the wellbeing of older adults depends primarily on their personality (Dumitrache et al., 2018), coping (Ren et al., 2021), spirituality (Sancho et al., 2020), character strengths (Baumann et al., 2020), and wisdom (Ardelt, 2016; Ardelt et al., 2023). These psychological variables contribute to one's meaningful and satisfying life and are more important for wellbeing than objective circumstances (Ardelt, 2003; Ardelt and Pridgen, 2022). In particular, wisdom achieved in later life demonstrated a substantial relationship with wellbeing (Ardelt, 2016; Ardelt and Jeste, 2022).

### Wisdom and wellbeing

Wisdom is considered to be a hallmark of psychosocial maturity (Taylor et al., 2011; Webster and Heintz, 2023). It might be operationalised as a multidimensional personality construct (Jeste et al., 2010; Ardelt et al., 2019; Ardelt and Pridgen, 2022; Webster, 2022) that allows individuals to apply their critical life experiences to facilitate the optimal development of the self and others (Webster, 2007, 2019). Wisdom embraces three main dimensions: cognitive, reflective, and affective (Ardelt, 2003, 2011) or—as Webster (2007) proposed—five components: critical life experiences, emotion regulation, reminiscence, openness, and humor. As a result, wiser people recognize and appreciate rich and varied experiences, are able to accept and regulate the full spectrum of human emotions, are more reflective, open to multiple perspectives, and use humor as a mature coping strategy. All these personality components may help people to maximize positive affect, to evaluate their life as good and meaningful, to self-realize in different aspects of life, and—as a result—to achieve greater wellbeing.

Wisdom has been shown to have various associations with wellbeing in many studies (Zacher and Staudinger, 2018; Indumati and Kenchappanavar, 2023). Studies to date have shown, among other things, that wisdom is positively related to both PWB (Glück et al., 2013; Wink and Staudinger, 2016) and SWB (Ardelt, 2000; Le, 2011; Taylor et al., 2011; Bergsma and Ardelt, 2012; Grossmann et al., 2013; Krause and Hayward, 2015; Krause, 2016; Shi et al., 2016), also in the long term (Ardelt, 2016). Moreover, some studies have revealed the possible mechanisms linking wisdom and indicators of wellbeing, such as coping strategies, perceived control, life engagement (Etezadi and Pushkar, 2013), or purpose in life (Ardelt and Edwards, 2015). On the other hand, some studies failed to confirm the wisdom–wellbeing relationship (Mickler and Staudinger, 2008; Mansfield et al., 2010) or found some irregularity. For instance, Zacher et al. (2013) revealed that wisdom and life satisfaction were positively correlated, but their association was non-significant when emotional intelligence was controlled for.

The association between wisdom and wellbeing may be better understood by more knowledge of other potential mediating factors, for example, forgiveness. Scholars (Mickler and Staudinger, 2008; Zacher and Staudinger, 2018) claim that gains in wisdom may result from overcoming negative challenging life events, whereas forgiveness is one of such positive psychological responses to incidents (Wade and Worthington, 2003; Dortaj et al., 2021).

### Wisdom and forgiveness

Forgiveness involves a shift from negative emotions, thoughts, and behavior following being treated unjustly to neutral or positive ones (Toussaint and Friedman, 2009; Worthington, 2009; Worthington and Wade, 2019; Li et al., 2020). It involves the process of reframing perceived transgressions in such a way that the offended person's reactions are no longer negative (Thompson et al., 2005). Wise people's prosocial attitudes and behaviors, as well as their reflectiveness of life experiences and their role in events (Ardelt, 2003; Bluck and Glueck, 2004; Webster, 2007, 2019; Bangen et al., 2013; Takahashi, 2019), can help them understand transgressions in a more complex way and deal with episodes committed by others, themselves and situations beyond anyone's control (Thompson et al., 2005; Sternberg and Glück, 2019) by practicing forgiveness. Wisdom is a frequently theorized but rarely tested factor related to forgiveness (Eghbali et al., 2022).

To our best knowledge, only four studies (Taylor et al., 2011; Booker and Dunsmore, 2016; Koshy et al., 2017; Eghbali et al., 2022) so far have explored relationships between wisdom and forgiveness. Although Booker and Dunsmore (2016) found no differences in forgiveness across four profiles of wisdom, Taylor et al. (2011) and Koshy et al. (2017) demonstrated a positive linkage between these variables. Eghbali et al. (2022) revealed that wiser people responded more prosocially and less antisocially after transgressions. A qualitative study by Choi and Landeros (2011) demonstrated that older people who were nominated as being wise attributed much importance to forgiveness, which they viewed as a way of coping with challenging life experiences. Thus, wisdom and forgiveness seem related.

### Forgiveness and wellbeing

On the other hand, forgiveness, as both a state and a trait, has been found positively related to indicators of wellbeing (e.g., Hill and Allemand, 2011; Ramírez et al., 2014; Kaleta and Mróz, 2018; Pizarro-Ruiz et al., 2021; Fincham and May, 2022). Two in-depth meta-analyses (Fu et al., 2016; Gao et al., 2022) proved that forgiveness was linked to subjective wellbeing. Being forgiving in a given hurtful event or across situations helps individuals to reduce their negative affect and increase positive one, as well as perceive themselves, other people, and life in a more positive way (Fincham et al., 2004; Kaleta and Mróz, 2021), which embrace elements of SWB (Diener et al., 2018). Forgiveness might also predict psychological wellbeing because forgiving individuals are able to reframe perceived transgressions and their consequences from negative to neutral or positive (Thompson et al., 2005). Not only does forgiveness restore their self-acceptance and positive relationships with the offenders, but it may also increase their sense of autonomy, mastery, purpose in life, and personal growth (Hill and Allemand, 2010; Kaleta and Mróz, 2018), which are all components of PWB.

**Figure 1:**
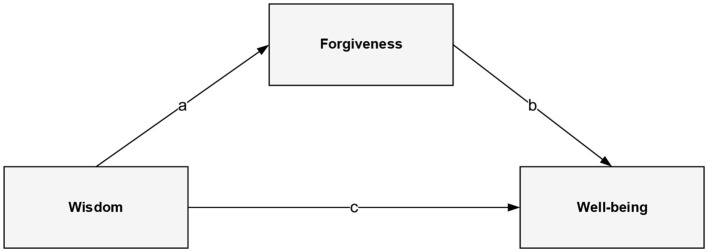
Model of the direct and indirect effects of wisdom on wellbeing through forgiveness.

Although wisdom, forgiveness, and wellbeing seem associated, they have scarcely been explored. Taylor et al. (2011) observed positive correlations between wisdom, trait forgiveness, and PWB. Koshy et al. (2017) found that individuals with high wisdom scores reported significantly greater trait forgiveness and happiness when compared to participants with low wisdom scores. To date, no study has proposed an advanced model of the relationships between the variables or tested the possible linking mechanisms. Thus, the first aim of the present study was to explore the relationships between wisdom, trait forgiveness, and wellbeing, both subjective and psychological. The second goal was to examine the mediating role of forgiveness in the association between wisdom and wellbeing (see Figure 1). In relation to the above, we made the following hypotheses:

**Hypothesis 1**. *Wisdom is positively associated with forgiveness and wellbeing (both subjective and psychological) in older adults*.
**Hypothesis 2**. *Forgiveness mediates the relationship between wisdom and wellbeing (both subjective and psychological) among older adults*.

## Methods

### Procedure

Older adults from different regions of Poland were recruited using the snowball sampling method (that is, every participant could recommend participation in the study to friends, acquaintances, or family members) to take part in a cross-sectional study. It means that the method applied reduced the representativeness of the sample. This is because participants tend to recruit people they know well, and therefore, people taking part in the study may have similar characteristics. This, in turn, means that the resulting sample may be a small subgroup of the general population. The study was conducted from October to December 2021. Data were collected using questionnaires. A set of instruments was prepared which included the following: (1) general instructions explaining the purpose of the study, (2) a personal inquiry form containing questions related to sociodemographic data, and (3) four questionnaires measuring the investigated variables. All the respondents were briefed on the study procedure and informed that participation was voluntary. The participants completed the tests at their own pace at home. A total of 503 sets of filled-in questionnaires were returned. Of these, 481 sets of questionnaires were used in the final analyses; the remaining 22 sets were either incomplete or incorrectly completed. The research procedure used received a positive opinion from the Ethical Committee at the Institute of Psychology of the John Paul II Catholic University of Lublin, Poland.

### Measures

#### Wisdom

Wisdom was evaluated using the Self-Assessed Wisdom Scale (SAWS) by Webster (2007). This tool is used to measure wisdom understood as a multidimensional construct comprising the following five components: Critical Life Experiences (e.g., *I have overcome many painful events in my life*), Emotional Regulation (e.g., *I can freely express my emotions without feeling like I might lose control*), Reminiscence-Reflection (e.g., *I often find memories of my past can be important coping resources*), Humor (e.g., *At this point in my life, I find it easy to laugh at my mistakes*), and Openness (e.g., *I enjoy sampling a wide variety of different ethnic foods*). The original SAWS consists of 40 items related to these areas of wisdom. The participants rate each item on a 6-point scale where 1 is “strongly disagree” and 6 is “strongly agree”. The SAWS has acceptable psychometric properties. Cronbach's α reliability coefficient^1^ for the global scale is 0.90, and the coefficients for the individual subscales range from 0.68 (Openness) to 0.88 (Reminiscence and Reflectiveness) (Webster, 2007; Taylor et al., 2011). In the present research project, a Polish version of the Scale, translated and adapted by Brudek (2022), was used. The internal consistency of the Polish Scale (α = 0.92) and its individual dimensions (from α = 0.60 to α = 0.84), as measured by Cronbach's α, are sufficient. The relatively low-reliability index for the “Openness” subscale may be worrying. However, the present study was based on the overall score on the scale in the area of wisdom and the reliability coefficient for the global scale was α = 0.94.

#### Forgiveness

Forgiveness was measured using the Polish adaptation (Kaleta et al., 2016) of Thompson et al.'s (2005) Heartland Forgiveness Scale (HFS). The HFS contains 18 items rated on a scale of 1 to 7 where 1 means “almost always false of me” and 7—“almost always true of me.” The scale characterizes forgiveness both at a general level and with regard to the following three dimensions: Forgiveness of Self (e.g., *Learning from bad things that I've done helps me get over them*), Forgiveness of Others (e.g., *Although others have hurt me in the past, I have eventually been able to see them as good people*), and Forgiveness of Situations (e.g., *I eventually make peace with bad situations in my life*). The psychometric properties of the Polish adaptation are satisfactory. Cronbach's α coefficient for the overall score was 0.87. In the present study, Cronbach's α coefficient for the entire scale was 0.74.

#### Psychological and subjective wellbeing

The Psychological Wellbeing Scale (Ryff and Keyes, 1995) was used to measure the level of individuals' development and self-realization. It consists of six subscales (42 items): Autonomy (e.g., *I am not afraid to voice my opinions, even when they are in opposition to the opinions of most people*), Environmental Mastery (e.g., *In general, I feel I am in charge of the situation in which I live*), Personal Growth (e.g., *I have the sense that I have developed a lot as a person over time*), Positive Relations with Others (e.g., *Most people see me as loving and affectionate*), Purpose in Life (e.g., *I have a sense of direction and purpose in life*), and Self-acceptance (e.g., *I like most aspects of my personality*). Items are rated on a 7-point Likert scale (1 = “strongly agree”; 7= “strongly disagree”). A global PWB score can be calculated by adding together the scores on the six dimensions. Cronbach's α coefficient for the overall score of the original version of the tool was 0.95. In the present study, we used the Polish version of the instrument, translated and adapted by Krok (2009). Cronbach's α coefficient for the current study was 0.89 for the global score.

A Polish version of the Satisfaction with Life Scale (SWLS; Juczyński, 2012) was used to evaluate global cognitive judgements of subjective wellbeing. SWLS is a popular and well-validated scale that represents the degree to which people are satisfied with their lives as a whole. The SWLS comprises five items (e.g., *In most ways, my life is close to my ideal*) rated on a 7-point Likert scale, ranging from 1 (“strongly disagree”) to 7 (“strongly agree”). A higher score indicates greater satisfaction with life. The alpha coefficient for the original version of the SWLS was 0.84. Cronbach's α for the present study was 0.84.

### Descriptive statistics

After preliminary analyses were conducted to test the common method bias, descriptive statistics were calculated for the investigated variables. The values of the descriptive statistics—means (*M*), standard deviations (*SD*), skewness (*ske*), and kurtosis (*k*)—of the scores obtained on each of the scales are shown in Table 1.

**Table 1:** Descriptive statistics and correlations between variables.

**Variables**	***M***	***SD***	***ske***	***k***	**Scores range**	**1**.	**2**.	**3**.	**4**.
1. Wisdom	4.30	0.68	−0.43	0.24	1–6	—			
2. Forgiveness	4.39	0.62	0.28	0.95	1–7	0.21^***^	—		
3. Subjective wellbeing (life satisfaction)	4.29	0.91	−0.09	−0.31	1–7	0.19^***^	0.39^***^	—	
4. Psychological wellbeing	4.53	0.62	0.16	0.85	1–7	0.35^***^	0.53^***^	0.48^***^	—

^***^p < 0.001.

First, it should be noted that the skewness and kurtosis values for all the variables were in the range of 1, −1, which indicates that the distribution of these variables did not deviate significantly from the normal distribution. Second, the mean wisdom, forgiveness, and wellbeing scores were above the mid-point of the rating scale. This means that the participants were characterized by above-average levels of wisdom and forgiveness and declared above-average wellbeing (see Table 1).

### Data analysis strategy

Given that all the variables were measured using self-report questionnaires, we assumed that the study could be biased by common method variance (Podsakoff et al., 2012). To verify this assumption, we used Harman's single-factor test. The calculations showed that one factor explained 15.15% of the variance, which was clearly below the admissible threshold of 50% (Podsakoff et al., 2003). It was therefore concluded that the present data were not affected by the common method bias.

As the next stage of the statistical study, once descriptive statistics and correlation between variables had been calculated, mediation analyses were performed to see whether forgiveness mediated the relationship of wisdom with the hedonic and eudaimonic dimensions of wellbeing. To this end, we used *IBM SPSS Statistics* 25.0 *PROCESS* macro 3.4. Model 4 was tested (see Figure 1). Mediation was assessed by evaluating the indirect effects between wisdom and (hedonic and eudaimonic) wellbeing. Standard errors for indirect effects were bootstrapped (10,000 samples) to provide a more accurate evaluation of the mediation tests. An indirect effect was considered statistically significant when the 95% coefficient interval (CI) for a coefficient did not include zero (Hayes, 2018).

## Results

### Participants

Data were gathered from 481 participants aged 60 to 92 (*M* = 68.84; *SD* = 6.31). The study included 312 women (64.9%) and 168 men. Surveys were carried out in various regions of Poland. Most of the respondents were inhabitants of urban areas. In total, 121 (121) individuals (25.2%) lived in large cities and 144 (29.9%) lived in small and medium-sized towns. The remaining respondents resided in the countryside (*n* = 194; 40.3%). In terms of the level of education, the most highly represented sample included persons with secondary education (*n* = 167; 34.7%). Among the respondents, 71 (71) (14.8%) had received primary education and 122 (25.4%) had vocational education. The remaining participants declared having higher education (*n* = 121; 25.2%). At the time when this research project was carried out, the vast majority of the respondents were retired (*n* = 398; 82.7%). Over half of the participants (*n* = 251; 52.2%) reported good self-rated health. In total, 145 (30.1%) rated their health as moderately good, whereas 85 respondents (17.7%) reported poor health. All participants were Roman Catholics.

### Correlations among variables

Correlations among wisdom, forgiveness, and wellbeing were examined. As hypothesized (H1), wisdom was positively associated with forgiveness and two dimensions of wellbeing—hedonic and eudaimonic. In the case of wisdom and life satisfaction, the relationship was weak. It was the same with psychological wellbeing. Moreover, moderate positive correlations were found between forgiveness and the hedonic and eudaimonic dimensions of wellbeing (see Table 1).

### Mediation analyses

**Figure 2:**
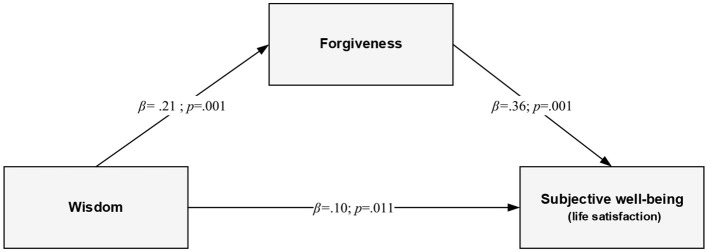
The final mediational model of the relationship between wisdom and subjective wellbeing (life satisfaction) through forgiveness (standardized coefficients).

**Figure 3:**
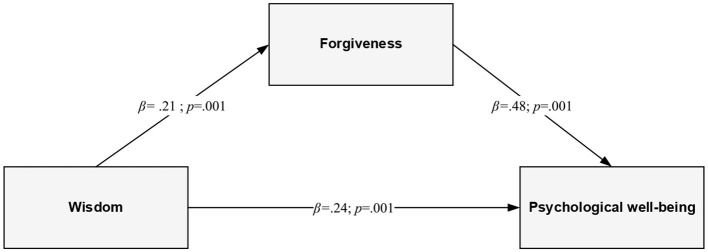
The final mediational model of the relationship between wisdom and psychological wellbeing through forgiveness (standardized coefficients).

Mediation analysis was performed to test whether forgiveness mediated the relationship between wisdom and the two dimensions of wellbeing—hedonic and eudaimonic. Table 2 contains the (non-standardized) regression coefficients of the model of mediation of the relationship of wisdom (X) with life satisfaction (Y_1_) and psychological wellbeing (Y_2_) by forgiveness (M). Statistically significant coefficients were obtained for paths describing the relationship between wisdom and forgiveness as well as the association between forgiveness and two dimensions of wellbeing—life satisfaction and psychological wellbeing. These results suggest that wisdom reinforces the tendency to forgive, which, in turn, is positively associated with both hedonic and eudaimonic wellbeing. The relationships are shown in graphical form in Figures 2, 3. For life satisfaction, the statistical analyses showed the significance of three effects: (1) a positive indirect effect (IE = 0.02, SE = 0.01; CI: 0.01; 0.03), (2) a positive direct effect (DE = 0.02, *SE* = 0.01; 95% CI: 0.01; 0.04), and (3) a positive total effect (TE = 0.04, *SE* = 0.01; 95% CI: 0.01; 0.04)^2^. The situation was similar in the case of psychological wellbeing. The following effects turned out to be statistically significant in the assumed mediation model: (1) a positive indirect effect (via forgiveness) (IE = 0.10, *SE* = 0.03; 95% CI: 0.05; 0.15), (2) a positive direct effect (DE = 0.23, *SE* = 0.04; 95% CI: 0.16; 0.31), and (3) a positive total effect (TE = 0.33, *SE* = 0.04; 95% CI: 0.25; 0.41). The indirect effects were small. For both the hedonic and the eudaimonic dimensions of wellbeing, the 95% CI did not include zero, which confirmed the mediation effects (Hayes, 2018). In addition, the mediation effects were confirmed by the Sobel test (*z*_LS_ = 4.14; *p* < 0.001; *z*_PWB_ = 4.45; *p* < 0.001).

**Table 2:** Regression coefficients, standard errors, and model summary information for the models tested.

**Antecedent**	**Consequent**								
	***M*** **(F)**			*Y*_1_ **(LS)**			*Y*_2_ **(PWB)**		
	***B***	***SE***	***p***	***B***	***SE***	***p***	***B***	***SE***	***p***
X (W)	0.09	0.02	0.001	0.02	0.01	0.011	0.23	0.04	0.001
*M* (F)	—	—	—	0.18	0.02	0.001	1.12	0.09	0.001
Constant	64.11	3.19	0.001	33.07	2.05	0.035	61.16	8.42	0.001
	*R*^2^ = 0.05			*R*^2^ = 0.16			*R*^2^ = 0.34		
	*F*_(1.479)_ *=* 22.59; *p* < 0.001			*F*_(2.478)_ *=* 45.75; *p* < 0.001			*F*_(2.478)_ *=* 123.59; *p* < 0.001		

W, wisdom; F, forgiveness; LS, life satisfaction; PWB, psychological wellbeing.

## Discussion

The present study aimed at exploring the relationships between wisdom, trait forgiveness, and wellbeing in older adults. The results revealed positive associations between the variables. The greater the wisdom the participants showed, the stronger the tendency to forgive and the better wellbeing they reported. This is in line with prior research on wisdom—wellbeing (Zacher and Staudinger, 2018) and forgiveness—wellbeing relationships (Davis et al., 2015; Webb and Toussaint, 2020; Gao et al., 2022). Wisdom helps people to have greater control over their life through active rather than passive coping (Choi and Landeros, 2011; Ardelt and Jeste, 2018; Ren et al., 2021) and deriving lessons from their life experiences (Glück and Bluck, 2013). Wise people are also able to accept life's unpredictability and uncertainties with calmness (Ardelt and Ferrari, 2014; Kunzmann and Glück, 2019). These all result in their greater sense of autonomy, environmental mastery, and purpose in life (Ardelt, 2016), aspects of psychological wellbeing (Ryff, 1989). Their deeper understanding of life also reduces stress and negative affect and increases subjective wellbeing (Rezaei and Jeddi, 2020).

Our study demonstrated that tendency to forgive mediated the positive relationship between wisdom and the wellbeing of older people. Thus, the results have not only confirmed simultaneous relationships between the variables (like Taylor et al., 2011; Koshy et al., 2017) but also suggested the mechanism in a sample of older individuals. Wise people, when confronted with life's demands and interpersonal incidents, are more willing to forgive and restore their positive regard for life, themselves, and others (Ardelt, 2003; Webster, 2007; Worthington, 2019), which, in turn, contributes to participants' wellbeing. A similar mediating role of forgiveness for wellbeing as the outcome variable has been previously shown. Tendency to forgive mediated in the relationship between religious struggle and satisfaction with life (Zarzycka, 2019), between marital adjustment, life satisfaction, and happiness (Kermani Mamazandi et al., 2020), and between real-life interpersonal hurts and psychological wellbeing (Gismero-González et al., 2020). Thus, trait forgiveness plays a mediating role in the relationships between different variables and wellbeing, both subjective and psychological.

This path might be particularly important for older adults. First, people become more forgiving with age (Toussaint et al., 2001; Steiner et al., 2011; Kaleta and Mróz, 2018). As older adults have experienced interpersonal problems and difficult situations throughout their lives, they have likely gained knowledge on the best ways of dealing with them (Charles and Carstensen, 2010). Older people could have learned that forgiveness is an especially constructive method and use it more frequently than younger individuals (Derdaele et al., 2019). Second, being in the final stage of life, aged individuals look back on their lives and attempt to accept the way things have turned out (Krause and Ellison, 2003). According to Erikson (1982), older people can achieve integrity (contrary to despair) if they see themselves as leading a successful life. A greater capacity to overcome resentment throughout one's lifetime would help one to keep calm, while positive forgiveness would promote positive feelings, such as pleasure, gratefulness, happiness, and overall satisfaction with life. Achieving integrity later in their life means appreciating themselves and others for who they are (Hamachek, 1990). Derdaele et al. (2019) showed that older individuals' trait forgiveness was related to life satisfaction by finding a balance between integrity and despair. On the other hand, it is wisdom that might be an antecedent of forgiveness. Wise people have distance from themselves and can judge events from different perspectives. They display positive emotions and benevolence toward other people (Ardelt, 2003; Webster, 2007), which helps them to deal with different transgressions through forgiveness. The link may be of importance for older rather than for other age groups as—according to gerotranscendence theory (Tornstam, 2005) and socioemotional selectivity theory (Carstensen et al., 1999)—high-quality relationships and maximizing positive emotions become more important as we age. Previous studies (Hebl and Enright, 1993; Lawler-Row and Piferi, 2006; Kaleta and Mróz, 2018) showed that trait forgiveness, especially positive forgiveness, has been significantly related to life satisfaction in late adulthood. Research also demonstrated that adults who forgive others tend to enjoy a greater sense of PWB than those who are less willing to forgive offenses (Gismero-González et al., 2020).

### Limitations

This study has some limitations. The first of these is its cross-sectional design, meaning that conclusions cannot be drawn regarding directionality. Only longitudinal research may prove that wisdom increases the tendency to forgive, which, in turn, affects the wellbeing of older individuals. Investigating the effects of forgiveness on wellbeing over time may not only provide further support for the findings of this study but also contribute to a better conceptualization of the relationship between forgiveness and wellbeing. In addition, the recruitment rate was not calculated. The second limitation affecting our research is that all the instruments used in this study were self-reporting measurement tools. This implies relying on participants to accurately evaluate and honestly report their responses. Third, our study focussed on the total score of forgiveness, which is a multidimensional construct. Future research could further investigate whether different types of forgiveness have the same associations with our outcome variables. For example, among older adults, forgiveness of others was positively associated with life satisfaction (Toussaint et al., 2001; Krause and Ellison, 2003), but no significant relationship was observed between self-forgiveness and life satisfaction (Toussaint et al., 2001). Finally, lack of control of variables such as health (physical and mental) status, status of residence (independent or nursing homes), social, and/or instrumental daily support prevents the generalization of results in the older population.

## Data availability statement

The original contributions presented in the study are included in the article/supplementary material, further inquiries can be directed to the corresponding author.

## Ethics statement

The studies involving humans were approved by the Ethical Committee at the Institute of Psychology of the John Paul II Catholic University of Lublin, Poland. The studies were conducted in accordance with the local legislation and institutional requirements. The participants provided their written informed consent to participate in this study.

## Author contributions

PB developing an idea for a research project, planning and conducting study, statistical data analysis, preparation of the initial version of the text (methodological part, results), and developing the final part of the text. SS co-editing and improving the prepared draft of the manuscript. KK collecting literature and preparation of the initial version of the text (theoretical introduction, discussion). All authors contributed to the article and approved the submitted version.

## Footnotes

^1^Cronbach's alpha coefficient measures the internal consistency, or reliability, of a set of survey items. It uses this statistic to help determine whether a collection of items consistently measures the same characteristic. Cronbach's alpha quantifies the level of agreement on a standardized 0 to 1 scale. Higher values indicate higher agreement between items. High Cronbach's alpha values indicate that response values for each participant across a set of questions are consistent. For example, when participants give a high response for one of the items, they are also likely to provide high responses for the other items. This consistency indicates the measurements are reliable and the items might measure the same characteristic (see Tavakol and Dennick, 2011).

^2^The abbreviations: IE, DE, and TE stand for effect size for indirect effect, direct effect, and total effect, respectively.
